# Perceived discrimination, health and mental health among immigrants in Norway: the role of moderating factors

**DOI:** 10.1186/s12889-019-6649-9

**Published:** 2019-03-20

**Authors:** Melanie Lindsay Straiton, Arild Kjell Aambø, Rune Johansen

**Affiliations:** 1 Department of Mental Health and Suicide, Division for Mental and Physical Health, Norwegian Institute of Public Health, PO Box 222, Skøyen, 0213 Oslo, Norway; 2 Unit for Migration Health, Division for Health Services, Norwegian Institute of Public Health, PO Box 222, Skøyen, 0213 Oslo, Norway

**Keywords:** Migrant health, Mental health, Discrimination, Ethnic identity

## Abstract

**Background:**

Ethnic discrimination is a relatively common experience among immigrants and ethnic minorities. The experience of discrimination can have detrimental effects on an individual’s health and well-being. This study investigated the association between perceived discrimination and general health and mental health among immigrants in Norway, in order to identify potential protective factors.

**Methods:**

Using data from the *Living Conditions Survey among Immigrants 2016*, our sample consisted of 4294 participants aged 16–66 years from 12 different countries. Participants were asked about a variety of themes including health and mental health, perceived discrimination, sense of belonging and language proficiency.

**Results:**

Around 27% of participants reported perceived discrimination. While perceived discrimination was not associated with general health, logistic regression analyses indicated that it was associated with 1.86 higher odds of mental health problems, even after adjusting for sociodemographic and psychosocial variables. Further, interaction analyses suggested that sense of belonging and trust in others moderated the relationship. Those with higher levels of trust did *not* have increased odds of mental health problems when experiencing discrimination, while those with low levels of trust did. In line with rejection sensitivity theory, the association between perceived discrimination and mental health was stronger for participants who had a strong sense of belonging to their own country of origin but not to Norway compared with those who had a sense of belonging to both.

**Conclusions:**

Improved integration strategies could potentially improve the mental health of immigrants as well as increase the acceptability of diversity, which in turn, could reduce discrimination towards immigrants. Limitations and suggestions for further research are discussed.

## Background

Immigration to and within Europe has increased substantially in the last few decades [1]. Studies show that migration may be a risk factor for mental health problems [2] and that some groups of migrants may also develop poorer health than the receiving country’s general popu lation over time [3]. A range of pre- and post-migration factors appear to contribute to this increased risk [4, 5]. Perceived discrimination is one of them. The aim of this study was to investigate the relationship between perceived discrimination and two health outcomes: self-perceived general health and mental health, and possible protective factors, among immigrants in Norway.

In 2018, immigrants made up around 14% of the population in Norway [6]. In 2000, they made up only 6% [7]. Polish immigrants are by far the biggest group and make up around 13% of all immigrants. The biggest groups from countries outside of the European Union now include Somalia, Syria, Iraq, Eritrea, The Philippines, Pakistan, Thailand, Iran and Afghanistan [6]. Since 1990, around one in three immigrants have come for work, one in three for family reunification, one in five for protection and one in 10 to study [8]. We have little knowledge of the extent to which immigrants in Norway experience discrimination.

Discrimination is the unfair treatment of a person or group due to particular characteristics such as gender, age, ethnicity, sexual orientation or disability [9]. Perceived discrimination (PD) is when people themselves perceive or experience discrimination [10]. This may include events that are not discriminatory according to the law or scientific definitions. Similarly, it can exclude events that are discriminatory by law or scientific definition if they are not experienced as such by the person in question [10]. PD can be at the institutional level, or the personal level and can manifest itself in both blatant and subtle forms [11]. It may or may not be intentional.

PD is associated with an increase in depression, anxiety and psychological distress as well as a decrease in well-being [12, 13]. Additionally, the more frequent the PD, the greater the risk of mental health problems [14, 15]. Repeated exposure to PD is also associated with poorer health on a range of different outcomes including self-perceived health, chronic health problems and even mortality [13, 16–18]. Studies focusing on perceived ethnic discrimination in particular, show that these associations are pervasive across different groups of immigrants and ethnic minorities, including Latinos, Asians and African-Americans in the USA [19–21] and various different immigrant groups in Europe [14, 20, 22]. A study of immigrant workers in Spain estimated that up to 40% of cases where immigrants reported a deterioration in health since migration were attributable to PD due to immigrant status [16].

However, PD is a stressor, and the effect of stress on health and illness may depend on one’s resources, personality and ability to cope [23]. In other words, the association between PD and health and mental health can depend on whether certain other conditions are present. Research suggests a number of factors may buffer the relationship between PD and health and mental health, though findings are dependent on the setting and the group studied. While high socioeconomic position, for instance, may protect against the effect of PD on mental health for Latino adolescents [24], among African American youth, those with higher socioeconomic position appear more vulnerable to the negative effects of PD [25]. A meta-analysis also revealed mixed results for social support [26]. While some of the included studies in the meta-analysis found that high social support related to a weaker effect of PD on mental health, the authors found that in most studies, social support only had a universal effect on mental health and health [26]. A Norwegian study among adolescents found that the role of social support differed with ethnic background [27].

Religion can be another way to help cope with stress and can provide a sense of resilience. Studies from the USA indicate that, among African Americans, attendance of religious ceremonies may buffer against the effects of PD on mental health [28], and that those who report high spirituality are less susceptible to psychological distress than those with low spirituality in the face of discrimination [29]. Another form of resilience may be strong ethnic identification, which is associated with higher self-esteem and lower levels of depression [30]. Identifying with a group may help individuals feel more connected to others, allowing them to focus on positive aspects of their group after experiencing PD [15]. A study among Asian Americans however, found that those who only identified with their own ethnic group were actually more susceptible to negative health effects of PD [31]. Acculturation theory suggests that individuals who have a strong orientation towards both their own group and the new culture may experience better levels of mental health than those who only feel they belong to one, or neither group [32].

Limited language proficiency is associated with poorer health and mental health [33, 34]. The combination of poor language proficiency and the experience of discrimination is related to greater use of informal mental health services among Asian Americans in the US [35]. Whether good language proficiency can protect against poor mental health in the face of discrimination is, to our knowledge, unknown. A final possible moderator is trust in others. Trust in others is a form of social capital and high levels of trust are potentially associated with good health and mental health [36]. At the same time, among depressed people, a higher level of social capital is associated with lower perceived discrimination [37]. A Swedish study found that generalised trust may confound the relationship between anticipated discrimination and mental health [38]. However, it is probable that high levels of trust could also protect against the effects of PD on mental health.

The association between PD and mental health and health is likely to, not only vary with the resources the person has available to cope with PD, but also the context (including the country) in which PD occurs [14]. The aim of the current study was to shed more light on the relationship between perceived discrimination and health and mental health among immigrants in Norway, and to identify possible protective factors. While immigrants may experience multiple types of discrimination, such as discrimination due to gender, age, sexual orientation or disability, we focused only on PD due to immigrant background. More specifically, we investigated:

1. Whether immigrants who reported perceived discrimination due to their immigrant background were at higher risk of mental health problems and poorer health than immigrants who did not.
2. If sociodemographic and psychosocial factors could explain the relationship.
3. If social support, trust in others, financial situation, religiosity, language proficiency or sense of belonging moderated the relationship between perceived discrimination and health/mental health.

## Methods

### Dataset

We used data from Statistics Norway’s Living Conditions Survey for Immigrants 2016 [39]. This survey covered a wide range of topics including demographic factors, family, housing, employment and the working environment, social contact, discrimination, Norwegian proficiency, attitudes and values and health. Many of the questions are based on questions in the European Social Survey [40], in addition to some migration specific questions. The survey included immigrants from 12 countries: Poland, Turkey, Bosnia-Herzegovina, Kosovo, Eritrea, Somalia, Afghanistan, Sri-Lanka, Iraq, Iran, Pakistan and Vietnam. These groups are among some of the largest, or rapidly growing, immigrant groups in Norway. At the time of data collection, there were 214,000 immigrants from these countries living in Norway, making up almost one third of all immigrants [39].

This dataset is an anonymised dataset collected by Statistics Norway and issued by the Norwegian Centre for Research Data. Because the dataset was anonymised, specific ethical approval or consent from participants was not required for this study. We conducted the analyses in accordance with the Norwegian Centre for Research Data’s data protection regulations.

Participation criteria included being an immigrant, being over the age of 16 and having lived in Norway for two or more years. Immigrants were defined as individuals born abroad with two foreign-born parents and four foreign-born grandparents [41]. Statistics Norway randomly selected 8156 immigrants from the 12 countries who met participation criteria and invited them to participate in the survey. Data were collected via computer assisted face-to-face and telephone interviews between November 2015 and July 2016. Interviews were available in each of the 12 countries main language(s) in addition to English. For a more in-depth description of sampling and data collection please see the report from Statistics Norway [39].

Overall, 4435 participants took part in the survey, yielding a response rate of 54.3%. In the current study, we excluded participants aged 67 years or more (retirement age) (*N* = 85). A further 58 participants (1.3%) were missing data on discrimination and were excluded in the analysis. Our overall sample therefore consists of 4294 participants; 2343 men (54.6%) and 1951 women (45.4%). This reflects the actual gender distribution of immigrants from the 12 included countries [39]. Table 1 shows the number of participants in each of the 12 immigrant groups, together with the gender and age distribution.

**Table 1:** Number and percentage of participants by country of origin, gender and age group

	Men N (%)	Women N (%)	Total N (%)
Country of origin			
Poland	242 (10.3%)	125 (6.4%)	367 (8.5%)
Turkey	180 (7.7%)	163 (8.4%)	343 (8.0%)
Bosnia-Herzegovina	182 (7.8%)	166 (8.5%)	348 (8.1%)
Kosovo	203 (8.7%)	165 (8.5%)	368 (8.6%)
Eritrea	190 (8.1%)	192 (9.8%)	382 (8.9%)
Somalia	181 (7.7%)	179 (9.2%)	360 (8.4%)
Afghanistan	244 (10.4%)	111 (5.7%)	355 (8.3%)
Sri Lanka	198 (8.5%)	176 (9.0%)	374 (8.7%)
Iraq	179 (7.6%)	162 (8.3%)	341 (7.9%)
Iran	209 (8.9%)	176 (9.0%)	385 (9.0%)
Pakistan	186 (7.9%)	156 (8.0%)	342 (8.0%)
Vietnam	149 (6.4%)	180 (9.2%)	329 (7.7%)
Age group			
16–24	339 (14.5%)	234 (12.0%)	573 (13.3%)
25–44	1160 (49.5%)	1154 (59.1%)	2314 (53.9%)
45–66	844 (36.0%)	563 (28.9%)	1407 (32.8%)
Total	2343 (100.0%)	1951 (100.0%)	4294 (100.0%)

### Variables

#### Mental health problems

Mental health problems were measured using the 5-item Hopkins Symptoms Checklist (HSCL) [42]. This is a shortened version of the more widely used 25-item HSCL, an instrument for measuring symptoms of depression and anxiety which has been applied in different populations and cultures. The five-item version includes the following symptoms: *1) nervousness or shakiness inside 1) feeling fearful, 2) feeling hopeless about the future, 4) Feeling blue 5) worrying too much about things*. Participants were asked to indicate the extent to which each of the symptoms has bothered them in the last 14 days on a four-point scale: (1) not at all, (2) a little 3) quite a bit or 4) extremely. We calculated mean scores based on participants having answered at least four of the five questions. An average score over two indicated symptoms of a clinically significant level [42]. Those with scores > 2 were coded as having mental health problems while scores of 2 or lower were coded as not having mental health problems.

#### General health status

Participants were asked if they considered their health in general to be: a) very good, b) good, C) neither good nor poor d) poor e) very poor. Participants who rated their health as good or very good were grouped as having good health, while the others were grouped as not having good health.

#### Perceived discrimination (PD)

Participants were asked if they felt they had been treated differently in the last 12 months 1) in the workplace, 2) in educational institutes 3) in the health care system and 4) other situations. For each arena, participants indicated if they felt this was due to their immigrant background. While all participants were asked about ‘other situations’, they were only asked about the other three arenas if it was relevant for them, i.e. those who were working, were asked about the workplace, those who were studying were asked about educational institutes and those who had used health care services were asked about the health care system. Since not all questions were relevant to all participants, those who responded yes to experiencing discrimination in at least one of the four areas were coded as having perceived discrimination while all others, who had answered no to all relevant arenas were coded as not having perceived discrimination.

#### Sociodemographics

##### Gender

Man/Woman. Man was reference category.

##### Age group

16–24 years, 25–44 years and 45–66 years. Age 45–66 years was the reference category.

##### Country of origin

Poland, Turkey, Bosnia-Herzegovina, Kosovo, Eritrea, Somalia, Afghanistan, Sri-Lanka, Iraq, Iran, Pakistan and Vietnam. Vietnam was the reference category.

##### Self-reported financial situation

Participants were asked: *How easy or difficult is it for you/ your family to make ends meet based on your household’s income? Is it: extremely difficult, difficult, somewhat difficult, somewhat easy, easy, extremely easy.* Responses were coded from 1 to 6, where 6 indicates a better financial situation.

*Psychosocial variables.*

##### Social support

Participants were asked: *Is there someone you are close to who you can confide in?* (Yes/No). No was the reference category.

##### Norwegian proficiency

Measured on a 5-point scale with the question: *Would you say that your Norwegian proficiency is very good, quite good, okay, quite poor or extremely poor?* We reversed the scale so that a score of one indicated extremely poor language proficiency and five very good language proficiency.

##### Trust in others

Trust was measured on an 11-point scale by one question: *Would you generally say that most people can be trusted, or that you can never been too careful when it comes to others?* Scores ranged from 0 to 10 where 10 indicated a higher level of trust.

##### Sense of belonging

Participants were asked: *To what extent do you feel a sense of belonging to Norway?* And *To what extent do you feel affiliation with your country of origin*? Responses were on a scale of 1 to 7 where 1 indicated ‘no affiliation’ and 7 means ‘great affiliation’. Cut-off scores of 5 (which was the approximate mean for affiliation with country of origin) and above were coded as having a sense of belonging to Norway or to country of origin. Based on this, 4 categories for sense of belonging were developed: 1) to both (those with a score of 5 or more on both questions), 2) to Norway (those with a score of 5 or more for Norway, and lower than 5 for country of origin), 3) to country of origin (those with a score of 5 or more on country of origin and 4) to neither (those with scores below 5 on both measures). To both was the reference category.

##### Religiosity

This was measured on an 11-point scale (0–10): *How important would you say religion is in your life?* Higher scores indicate greater religiosity.

### Analyses

We used a combination of chi-square analyses, t-tests and Mann Whitney U to look at differences between those who reported PD, mental health problems or good health compared with those who did not. We then ran logistic regression analyses to assess the relationship between mental health and PD while controlling for covariates. Finally, we ran various logistic regressions with interaction effects between PD and social support, trust, language proficiency, sense of belonging and religiosity.

#### Missing data

Overall, only 1.5% of the data was missing. For Norwegian proficiency, however, we were missing 5.6% of the data. This was due to a programming problem Statistics Norway experienced during data collection (see Appendix for more information). Participants missing data on this variable were more likely to be from Eritrea (25%), Somalia (32%), Afghanistan (17%) or Iraq (13%) and had, on average, a poorer financial situation. Around three quarters were aged between 25 and 44 years. We were also missing 4.1% of data on financial situation. These participants were more often women (55%), more often in the youngest age category (63%) and more likely to be from Afghanistan (18%) or Pakistan (18%). This may indicate that participants missing data on financial situation did not have much responsibility for the household’s finances. Missing data on other variables was minimal.

Although our data were not missing completely at random, we replaced missing data with mean values for our continuous variables; financial situation, Norwegian proficiency, religiosity, sense of belonging (to Norway and to own group) and trust in others. Statistical experts suggest that when missing data is around 5%, more complex estimation methods for missing data will yield the same imputed values [43]. On the categorical variables, there were only 17 participants (0.04%) missing data on social support, 17 (0.04%) on mental health and seven (0.02%) on general health. These cases were excluded listwise.

## Results

### Characteristics of those who experience discrimination

PD due to immigrant status was reported by 26.5% (*n* = 1137) of participants. Table 2 shows the percentage who reported discrimination by the various sociodemographic and psychosocial variables. There were no significant gender differences but PD was more common in younger age groups; 36.3% for 16–24 years compared with 19.6% among 45–66 years. Those who experienced PD were more likely to have a poorer financial situation (3.45) than those who did not (3.61).

**Table 2:** Characteristics of those who have perceived discrimination, who have mental health problems and who have good general health status

	Perceived discrimination % (N)		Mental health problems % (N)		General health % (N)	
Total	26.5 (1137/4294)		12.7 (545 /4277)		71.4 (3060/4287)	
Gender	ns		^***^		^***^	
Men	27.0 (633)		10.8 (252)		74.1 (1733)	
Women	25.8 (504)		15.1 (293)		68.1 (1327)	
Age group	^***^		^***^		^***^	
16–24	36.3 (208)		11.2 (64)		87.6 (501)	
25–44	28.2 (653)		11.1 (256)		78.4 (1812)	
45–66	19.6 (276)		16.0 (225)		53.2 (747)	
Country of origin	^***^		^***^		^***^	
Poland	29.7 (109)		9.9 (36)		81.4 (298)	
Turkey	29.4 (101)		19.6 (67)		60.6 (208)	
Bosnia-Herzegovina	21.3 (74)		13.0 (45)		74.1 (257)	
Kosovo	24.5 (90)		13.1 (48)		73.1 (269)	
Eritrea	18.6 (71)		6.5 (25)		81.4 (311)	
Somalia	26.7 (96)		6.1 (22)		83.6 (300)	
Afghanistan	28.7 (102)		13.7 (48)		76.6 (271)	
Sri Lanka	12.8 (48)		7.2 (27)		61.8 (231)	
Iraq	38.7 (132)		19.9 (68)		64.2 (219)	
Iran	37.7 (145)		21.3 (82)		68.2 (262)	
Pakistan	29.2 (100)		15.9 (54)		65.4 (223)	
Vietnam	21.0 (69)		7.1 (23)		64.3 (211)	
Social support	ns		^***^		^***^	
Yes	26.3 (936)		11.2 (395)		73.8 (2620)	
No	27.1 (196)		20 (145)		59.8 (432)	
Sense of belonging	^***^		^***^		^**^	
To both	22.6 (529)		10.9 (253)		70.5 (1643)	
To Norway	27.8 (351)		13.6 (171)		74.7 (943)	
To country of origin	35.6 (176)		16.1 (79)		69.4 (343)	
Neither	40.7 (81)		21.2 (42)		65.8 (131)	
Perceived discrimination			^***^		ns	
Yes	–		20 (226)		69.7 (793)	
No	–		10.1 (319)		72.0 (2267)	
	Yes	No	Yes	No	Yes	No
	Mean (sd)	Mean (sd)	Mean (sd)	Mean (sd)	Mean (sd)	Mean (sd)
Financial situation (1–6)	3.45 (1.32)	3.61 (1.33)^***^	2.87 (1.41)	3.67 (1.28)^***^	3.74 (1.28)	3.13 (1.36)^***^
Trust in others (0–10)	5.62 (2.47)	6.22 (2.32)^***^	5.10 (2.69)	6.21 (2.30)^***^	6.21 (2.32)	5.71 (2.49)^***^
Norwegian proficiency (1–5)	3.90 (0.94)	3.75 (1.00)^***^	3.65 (0.99)	3.81 (0.99)^**^	3.90 (0.98)	3.53 (0.97)^***^
Religiosity (0–10)	6.45 (3.75)	6.73 (3.59)^*^	6.62 (3.71)	6.65 (3.62) ^ns^	6.53 (3.68)	6.94 (3.50)^**^

Ns: *p* > 0.05, ^*^p < 0.05, ^**^p < 0.01; ^***^p < 0.001

PD also varied substantially by country. The lowest proportion reporting discrimination was among Sri-Lankans (12.8%), while it was highest among Iraqi and Iranian Immigrants (37.7–38.7%).

While PD was not associated with social support, sense of belonging was. Those who had a sense of belonging to both Norway and their country of origin were the least likely to report discrimination (22.6%), while those with no sense of belonging were most likely (40.7%). Those reporting discrimination were less trusting of others and regarded religion as less important than those who did not report discrimination. Norwegian proficiency was higher among those reporting discrimination (3.90) compared with those not reporting discrimination (3.75).

### Characteristics of those with mental health problems and characteristics of those with good general health status

Analyses were based on 4278 for general health and 4277 for mental health. Overall, 12.7% (*n* = 545) scored above the cut-off for mental health problems and 71.4% (*n* = 3060) for good general health. Table 2 also shows the characteristics of those who reported mental health problems and those who reported good versus not good general health status. Women more often reported mental health problems than men (15.1% vs 10.8% respectively) and less often reported good health (68.1% vs 74.1%). A higher percentage of older participants (16.0%) reported mental health problems compared with the younger groups (approximately 11%). Age was also associated with poorer health, with only 53.2% in the oldest age group reporting good health compared with 87.6% in the youngest group. Participants with mental health problems also reported a substantially poorer financial situation, as did those who did not have good health, compared with those without mental health problems and good health.

As with PD, mental health problems and general health also varied substantially by country of origin. The lowest rates of mental health problems were among immigrants from Somalia, Eritrea, Sri-Lanka and Vietnam (6.1–7.2%) while the highest were among immigrants from Turkey, Iraq and Iran (19.6–21.3%). Immigrants from Poland, Eritrea and Somalia most often reported good health (81.4–83.6%) while this was least common among immigrants from Turkey and Sri-Lanka (60.6–61.8%).

The proportion of participants with mental health problems was almost double among those who reported PD (20.0%) compared with those who did not (10.1%). In contrast, there was no significant association between PD and general health status.

Mental health problems were more common among those without social support (20.0%) compared with those who had social support (11.2%). Good health was more common among those with social support (73.8%) than those without (59.8%). Participants with mental health problems also had lower levels of trust and poorer Norwegian skills compared to those with better mental health. Religiosity was not associated with mental health problems but sense of belonging was. The proportion with mental health problems was highest among those who did not feel they belonged to either Norway or their country of origin (21.2%), and lowest among those who felt they belonged to both (10.9%). Good general health was positively related to trust in others and Norwegian skills but negatively related to religiosity. The proportion with good health was lowest among those with no sense of belonging to either Norway or their country of origin (65.8%), while it was highest among those who only had a strong sense of belonging to Norway (74.7%).

### Can sociodemographic and psychosocial factors explain the association between PD and mental health problems?

Model 1 in Table 3 shows that the odds of having a mental health problem among those who reported PD compared with those who did not were reduced from 2.22 (model 1) to 2.03 (model 2) when we included sociodemographic variables. In model 3, we included psychosocial variables in the analysis except for religiosity and Norwegian proficiency since they did not relate to mental health problems in model 1 or in model 2. The odds of having mental health problems were 1.86 higher among those who reported PD compared to those who did not, after adjusting for all variables. Sociodemographic and psychosocial factors therefore explained some, but far from all of the relationship between PD and mental health.

**Table 3:** Odds ratio and 95% confidence intervals for mental health problems

	Model 1	Model 2	Model 3
PD	2.22 (1.84–2.67)^***^	2.03 (1.66–2.48)^***^	1.86 (1.51–2.28)^***^
Gender	1.46 (1.22–1.75)^***^	1.54 (1.27–1.87)^***^	1.64 (1.34–2.00)^***^
Age: 45–66	Ref	Ref	Ref
16–24	0.66 (0.49–0.89)^**^	0.67 (0.48–0.94)^*^	0.66 (0.47–0.94)^*^
25–44	0.66 (0.54–0.80)^***^	0.62 (0.50–0.76)^***^	0.62 (0.50–0.77)^***^
Country of origin			
Vietnam	Ref	Ref	Ref
Poland	1.44 (0.84–2.50)	1.68 (0.96–2.95)	1.52 (0.86–2.71)
Turkey	3.22 (1.95–5.32)^***^	2.68 (1.59–4.50)^***^	2.78 (1.64–4.72)^***^
Bosnia-Herzegovina	1.97 (1.16–3.34)^*^	2.25 (1.31–3.87)^**^	2.71 (1.56–4.71)^***^
Kosovo	1.98 (1.18–3.34)^*^	1.96 (1.11–3.28)^*^	2.34 (1.35–4.04)^**^
Eritrea	0.92 (0.51–1.66)	0.65 (0.36–1.19)	0.73 (0.40–1.35)
Somalia	0.86 (0.47–1.57)	0.51 (0.27–0.96)^*^	0.59 (0.31–1.13)
Afghanistan	2.09 (1.24–3.52)^**^	1.96 (1.13–3.41)^*^	1.98 (1.13–3.48)^*^
Sri Lanka	1.03 (0.58–1.83)	0.68 (0.38–1.23)	0.97 (0.53–1.77)
Iraq	3.28 (1.99–5.41)^***^	1.94 (1.15–3.28)^*^	2.09 (1.23–3.55)^**^
Iran	3.57 (2.19–5.81)^***^	2.66 (1.60–4.40)^***^	2.80 (1.67–4.68)^***^
Pakistan	2.49 (1.49–4.16)^**^	1.94 (1.14–3.30)^*^	2.23 (1.30–3.84)^**^
Sense of belonging			
To both	Ref		
To Norway	1.29 (1.05–1.59)^*^	1.38 (1.11–1.72)^**^	1.36 (1.08–1.70)^**^
To country of origin	1.57 (1.19–2.07)^**^	1.62 (1.20–2.18)^**^	1.38 (1.02–1.87)^*^
Neither	2.21 (1.54–3.18)^***^	2.03 (1.37–3.01)^***^	1.63 (1.02–2.45)^*^
Financial situation (1–6)	0.63 (0.59–0.68)^***^	0.61 (0.56–0.66)^***^	0.63 (0.58–0.68)^***^
Social support	0.51 (0.41–0.63)^***^	0.61 (0.48–0.77)^***^	0.64 (0.51–0.83)^***^
Trust in others (0–10)	0.83 (0.80–0.86)^***^	0.84 (0.81–0.88)^***^	0.86 (0.82–0.89)^***^
Religiosity (0–10)	1.00 (0.97–1.02)	–	–
Norwegian proficiency (1–5)	0.86 (0.79–0.94)^**^	0.93 (0.83–1.03)	–

Model 1: univariate analyses, model 2: each variable adjusted for gender, age, country of origin and financial situation; model 3: model 2 with adjustment for social support, trust in others, Norwegian proficiency and sense of belonging

^*^p < 0.05, ^**^p < 0.01; ^***^p < 0.001

The model also shows that all sociodemographic factors still related to mental health problems after controlling for other covariates. Lack of social support and trust in others were also significant predictors of mental health problems. Women were 64% more likely than men to experience mental health problems, and immigrants in the oldest age category were almost 60% more likely to experience mental health problems than immigrants aged 25–44 years. Financial situation was a strong predictor of mental health problems with a 0.64 decrease in odds for every one-unit increase in financial situation. Those without social support were over 50% more likely to report mental health problems than those who did have social support. Additionally, for every one-unit decrease in trust in others, the odds of reporting mental health problems increased by 16%. Compared with participants who felt a sense of belonging to both Norway and their country of origin, those who only felt affinity to one country had 37% higher odds and those who felt it to neither had 62% higher odds of mental health problems.

Since PD did not relate to general health status, we did not conduct further analyses.

### Interactions with perceived discrimination

**Fig. 1:**
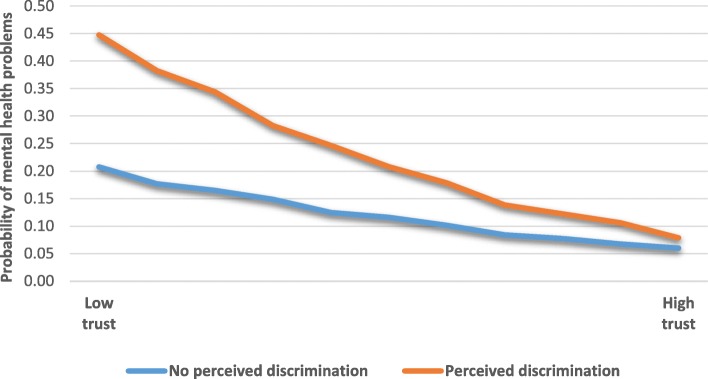
Interaction between perceived discrimination and trust in others for the probability of mental health problems

For mental health, we ran several logistic regression analyses to check for interaction effects between PD and social support, trust, sense of belonging and religiosity. In these analyses, we only controlled for gender and age in order to preserve power. We found two interactions (Table 4). The first was between PD and trust in others on mental health. The relationship between PD and mental health problems was stronger for those who reported low levels of trust than those who reported high levels. This relationship is displayed graphically in Fig. 1. We see that if one had high levels of trust, PD had little association with mental health, while among those with low levels of trust, PD was associated with higher odds of mental health problems. Thus, trust in others buffered in the relationship between PD and mental health.

**Table 4:** Interaction between perceived discrimination (PD) and trust in others and perceived discrimination and sense of belonging

	Mental health problems	
	β	OR (CI)
Percieved discrimination	1.22	3.39 (2.17–5.30)^***^
Trust in others	−0.15	0.86 (0.82–0.90)^***^
PD ^*^ trust in others	−0.08	0.92 (0.85–0.99)^*^
PD	1.52	4.57 (2.16–9.65)^***^
Sense of belonging		
To both	Ref	
To Norway	0.23	1.26 (0.97–1.64)
To country of origin	0.12	1.13 (0.75–1.69)
To neither	0.90	2.46 (1.49–4.08)^***^
PD ^*^ Sense of belonging – to both	Ref	
^*^To Norway	0.11	1.12 (0.72–1.73)
^*^To country of origin	0.62	1.87 (1.05–3.32)^*^
^*^To neither	−0.31	0.73 (0.35–1.56)

^*^p < 0.05, ^***^p < 0.001

**Fig. 2:**
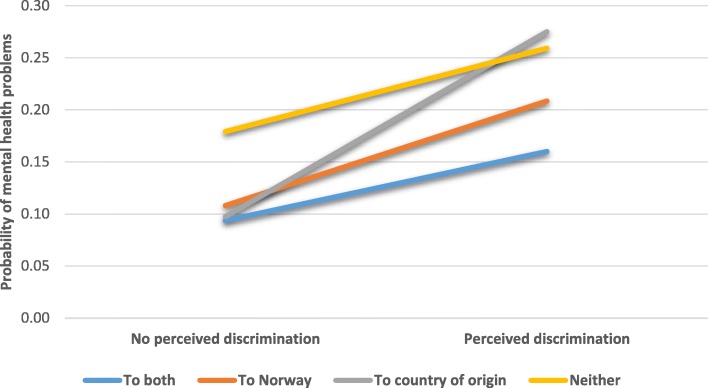
Interaction between perceived discrimination and sense of belonging for the probability of mental health problems

The second interaction was between PD and sense of belonging. Although all groups had higher odds of mental health problems in the face of discrimination, those with a sense of belonging to only their country of origin had a higher increase in odds than those who felt affiliation with both Norway and their country of origin. Thus, PD had a stronger relationship with mental health problems among those with a strong sense of belonging to only their country of origin. The relationship is displayed graphically in Fig. 2. Among those who did not experience discrimination, the probability of experiencing mental health problems was similar among those with a sense of belonging to at least one place, while those with no sense of belonging had a higher probability. However, in the face of discrimination, those who identified only with their country of origin had a much higher probability of mental health problems than those who had a sense of belonging to Norway or both Norway and their country of origin. In fact, the probability was as high as among the group without a sense of belonging to either country.

## Discussion

The aim of this study was to shed more light on the relationship between PD due to immigrant background and health and mental health among immigrants in a Norwegian context. Our study confirmed previous research showing a negative relationship between PD and mental health. Those who experienced discrimination at least once during the previous 12 months had 1.85 the odds of reporting mental health problems compared with those who had not experienced discrimination, even after controlling for various socio-demographic and psychosocial variables. Because of the cross-sectional nature of this study, we cannot be sure which direction the relationship goes in. There may also be underlying confounders that influence both, such as self-esteem, which we could not account for in this study. However, longitudinal studies tend to show support for PD affecting mental health rather than mental health predicting the subsequent reporting of discrimination [14, 44]. It is therefore likely that PD contributes to the mental health inequalities immigrants in Norway experience.

Previous studies also suggest an association between PD and general health status [13, 16, 18]. Yet, we found no difference in general health between immigrants who perceived discrimination and immigrants who did not. Research suggests that frequent PD can induce stress responses including increased blood pressure, heart rate, which through time can impact on health [26]. Frequent experiences can also lead to the engagement in poor health choices as a form of coping, such as unhealthy eating, smoking or alcohol abuse, which through time affect health. Another pathway is through a deterioration in mental health [14]. It is possible that one off events or occasional discrimination has less of an impact on health, at least in the short term. We were unable to determine the frequency of discrimination in this study. Thus, our study most likely underestimates the association between discrimination and health, as well as discrimination and mental health. There is therefore a need for detailed longitudinal Norwegian studies on discrimination among immigrants.

Another noteworthy finding is that younger immigrants included in our study were more likely to perceive discrimination than older immigrants. It may be that because younger immigrants tend to have higher levels of acculturation, they also have greater expectations of equal treatment and are thus more sensitive to picking up on potential episodes of discrimination [45]. Yet, despite younger people reporting more PD, and PD being associated with poorer mental health, the proportion reporting mental health problems was lower among younger immigrants, even after controlling for sociodemographic factors. This is in line with analyses of the previous Living Conditions Survey among immigrants, 2005/6, where the highest risk of mental health problems was found to be at around age 50 years [46]. It may be that other migration stressors influence the mental health of older immigrants’ more than younger immigrants. Interestingly, in the general population, risk for mental health problems is higher at a younger age [47].

In this study, we also investigated which factors buffered the relationship between PD and mental health. We found that sense of belonging not only had a main effect on mental health, but it also interacted with PD and mental health. Those with a sense of belonging to both Norway and their country of origin reported better mental health overall. This is in line with findings from acculturation research, where a meta-analysis showed that participants who adopt the language and behaviour of the new country, while simultaneously maintaining one’s ethnic identity, had advantages in terms of psychological well-being [48]. While overall, mental health was poorer among those who reported PD, we found that participants who only had a sense of belonging to their country of origin were more vulnerable to negative mental health effects of PD. This was unexpected, since many studies indicate that a sense of belonging with one’s ethnic group can attenuate the negative effects of PD [15]. Again, due to the cross-sectional nature of the study, we are unable to determine the direction of the relationship between these different variables. It may be that those with poorer mental health are also less likely to feel a sense of belonging to Norway. Nonetheless, our finding fits with rejection sensitivity theory, which suggests that those with a high sense of belonging to only their own country react more strongly to PD, since it is experienced as a total rejection of, not only oneself as a person, but also of the group with which they identify [31]. Thus, when facing discrimination, it may be advantageous to feel a sense of belonging to more than one group. Further research could investigate changes in sense of belonging over time and how this relates to both PD and mental health. It is possible that better integration strategies could not only improve integration but also improve mental health. Greater acceptance of diversity in Norwegian society may also reduce discrimination.

The second moderating factor we found was between trust in others and PD. Again, trust also had a main effect. It could be reasoned that if those who have poorer mental health are more likely to perceive discrimination, they will also be less trusting of others. By including trust in analyses, we were able to account for a general mistrust, which may make people more sensitive to discrimination. In the multivariate analyses, the odds of mental health problems increased from 1.86 to 1.96 when excluding trust from the analyses (not shown), suggesting that trust confounds a little of the relationship between PD and mental health. This is in line with findings from Sweden [38]. Nonetheless, the odds of mental health problems were still far higher among those who reported PD even after accounting for trust. Further, the interaction effect indicated little difference in mental health status between those who reported PD and those who did not among participants who had high levels of trust, while the difference was quite substantial for those with low levels of trust. This suggests that higher levels of trust may protect against the negative effects of PD. It is possible that those with higher levels of trust are able to minimise the PD experience, believing that the problem lies with the person doing the discrimination, and that people ‘in general’ do not discriminate. This way of compartmentalising may have less effect on mental health. However, it is also possible that trust could deteriorate with increased exposure to discrimination. More research on trust is required. Since trust relates to social capital, strategies that aim to increase social capital among immigrants could potentially be protective for mental health problems.

Social support is also a form of social capital but this factor did not moderate the relationship between PD and mental health. It only had a main effect, being associated with better mental health, as was being in a comfortable financial position. Strategies aiming to tackle social isolation or improve the socioeconomic situation of disadvantaged immigrants are likely to be beneficial for mental health.

Another interesting observation was that those with better language skills were more likely to report PD than those with poorer skills. Discrimination may often manifest in subtle forms and therefore those with poorer language skills may be less likely to pick-up on potential discrimination. On the other hand those who have good language proficiency are more likely to be working (and more exposed to the Norwegian society generally) and therefore be exposed more often to situations where discrimination may arise. We found, however, no association between language proficiency and mental health after controlling for sociodemographic factors. This is in contrast to previous research [33]. Language proficiency is positively related to education, labour market participation and socioeconomic status [49, 50], factors that are also associated with lower risk of mental health problems. Language proficiency may therefore be a general indicator of resources.

Finally, this study shows a striking similarity between PD and mental health problems by country of origin; several groups where a higher proportion reported PD often had a higher proportion with mental health problems. Accounting for discrimination and sociodemographic factors reduced the odds of mental health problems for Iranians from over 3.5 to 2.7 compared with Vietnamese for instance. There were some exceptions however. Somalis had low levels of mental health problems despite reporting high levels of PD. Unfortunately, in this study, we did not have enough power to investigate relationships within countries to see if different factors were protective among immigrants from different country backgrounds. Future research would benefit from larger sample sizes.

### Limitations

In addition to the limitations mentioned above about causality, sample size and our measure of PD, it is noteworthy that the response rate in the survey was only 54%. Statistics Norway indicated that some immigrants change address and mobile number often and others may leave the country without registering the emigration. On the other hand, the recent Living Conditions Survey among the general population yielded the same response rate [51] and immigrants are usually underrepresented in such population surveys. Thus, a response rate of 54% is considered acceptable. Nonetheless, selection bias is likely and we might expect that those with the poorest health and mental health choose not to participate in surveys. It is also possible that those who experience the most discrimination would be less inclined to participate. Thus, our findings may underestimate the link between health, mental health and PD.

The study was also not representative of all immigrants in Norway; around 46% of immigrants in Norway come from countries within the European Economic Area (EEA), yet only 8.5% of the participants in this survey came from a country within the EEA. Additionally, there are other large, non-EEA immigrant groups that are not included in this survey such as Syrians, Thai and Filipinos. Although far from representative of all immigrants in Norway, the gender and age distribution is fairly representative of each of the 12 included countries [39].

Another limitation is that this study focuses on PD related to having an immigrant background. We excluded one question about PD when job seeking. Although this is also likely to relate to mental health [52], we were interested in PD based on participation in society, rather than PD as a barrier to participation in society. Thus, our findings may not be directly comparable with others using the same dataset. Additionally, participants may also experience other types of discrimination that we have not accounted for such as gender, age or discrimination related to sexual orientation. It is well established that immigrant women report poorer mental health than immigrant men do but we found no gender difference in PD. It is possible that women experience double discrimination based on their gender, which may contribute to their higher rate of mental health problems.

## Conclusion

Overall, almost 27% of the participants in this study reported having experienced discrimination due to their immigrant background in the last year. Given the heightened odds of mental health problems for immigrants who report PD due to their immigrant background, we can speculate that PD contributes to health inequalities among immigrants. More longitudinal research is required in Norway in order to identify the mechanisms between PD and mental health. It is essential that we find new and better ways of tackling discrimination at the societal level. Strategies for improving social capital among immigrants, as well as ways of facilitating integration require more investigation.

Finally, as mentioned above, we were unable to determine the extent or the frequency of discrimination based on the questions in this survey. The next Living Conditions Survey among Immigrants should include a more comprehensive measure of PD in order to gain better insight into the relationship between PD and health and mental health. Further, samples focusing on a smaller number of immigrant groups, with a larger number of participants from each group, may give a more nuanced picture of the factors that relate most strongly to poor mental health among particular groups.

## Appendix

### Norwegian proficiency

Due to a programming mistake during data collection, immigrants who had taken part in an introduction program (offered to refuges and their family member since 2004) were not asked about their Norwegian proficiency. Statistics Norway attempted to obtain this information after data collection was completed. According to Statistics Norway, the missing information concerned around 17% of the original sample (753). Of these, around 50% had completed the interview in Norwegian. Statistics Norway managed to re-contact 550 of these participants to ask about language proficiency questions in Autumn 2016. During the time original participation and follow-up, Norwegian proficiency may have improved slightly. However, Statistics Norway believe this has would not substantially affect the findings. This explains why 5% were missing data on language proficiency and suggests that language proficiency is not missing at random. Participants who were missing language were mostly from Eritrea (26%), Somalia (33%), Afghanistan (18%) and Iraq (13%). Around three quarters were between 25 and 44 years and reported a poorer financial situation than the sample overall (3.02 vs 3.57).

## Abbreviations

EEA: European Economic Area
HSCL: Hopkins symptoms checklist scale
PD: Perceived discrimination
